# Key Modules and Hub Genes Identified by Coexpression Network Analysis for Revealing Novel Biomarkers for Spina Bifida

**DOI:** 10.3389/fgene.2020.583316

**Published:** 2020-12-02

**Authors:** Zijian Li, Juan Feng, Zhengwei Yuan

**Affiliations:** ^1^Department of Neurology, Shengjing Hospital of China Medical University, Shenyang, China; ^2^Key Laboratory of Health Ministry for Congenital Malformation, Shengjing Hospital of China Medical University, Shenyang, China

**Keywords:** spina bifida, weighted gene co-expression network analysis, bioinformatics analysis, pathological process, hub genes

## Abstract

Spina bifida is a common neural tube defect (NTD) accounting for 5–10% of perinatal mortalities. As a polygenic disease, spina bifida is caused by a combination of genetic and environmental factors, for which the precise molecular pathogenesis is still not systemically understood. In the present study, we aimed to identify the related gene module that might play a vital role in the occurrence and development of spina bifida by using weighted gene co-expression network analysis (WGCNA). Transcription profiling according to an array of human amniocytes from patients with spina bifida and healthy controls was downloaded from the Gene Expression Omnibus database. First, outliers were identified and removed by principal component analysis (PCA) and sample clustering. Then, genes in the top 25% of variance in the GSE4182 dataset were then determined in order to explore candidate genes in potential hub modules using WGCNA. After data preprocessing, 5407 genes were obtained for further WGCNA. Highly correlated genes were divided into nineteen modules. Combined with a co-expression network and significant differentially expressed genes, 967 candidate genes were identified that may be involved in the pathological processes of spina bifida. Combined with our previous microRNA (miRNA) microarray results, we constructed an miRNA–mRNA network including four miRNAs and 39 mRNA among which three key genes were, respectively, linked to two miRNA-associated gene networks. Following the verification of qRT-PCR and KCND3 was upregulated in the spina bifida. KCND3 and its related miR-765 and miR-142-3p are worthy of further study. These findings may be conducive for early detection and intervention in spina bifida, as well as be of great significance to pregnant women and clinical staff.

## Introduction

Spina bifida is one of the most common central nervous system (CNS) malformations found in fetuses: the neural tube shows incomplete closure, and is usually complicated by other neurological abnormalities and comorbidities such as hydrocephalus. Spina bifida can be broadly classified into two groups: spina bifida aperta and spina bifida occulta. The former refers to meninges or nerve tissue bulging out of the spinal canal through spina bifida and forms a cystic mass, so it also called spina bifida cystica, including myelomeningocele, meningocele, myelocele, etc. In general, there is no need to treat spina bifida occulta. Spina bifida mentioned in this study refers to spina bifida aperta. Spina bifida is often either treated after birth or an intrauterine defect repair is undertaken (Alabi et al., 2018). However, complications such as hydrocephalus, fecal dysfunction, voiding dysfunction, and limb mobility disorders cannot be completely avoided. To date, the etiology of spina bifida is not fully understood and may be related to genetic and environmental factors. Elucidation of the pathogenesis of spina bifida is urgently required in order to reduce harm to the fetus, as well as that to society and the family.

Low folic acid levels in pregnant women, or antiepileptic drugs such as valproic acid taken during pregnancy, and a history of obesity and diabetes in pregnant women are environmental risk factors for the disease. Folic acid supplementation in pregnant women, a prenatal diagnosis, and fetal *in utero* therapy can effectively reduce the number of children born with birth defects (Molloy et al., 2017). Evidence has shown that in spite of sufficient folic acid intake by pregnant women, impaired uptake and utilization of folic acid can also contribute to neural tube malformation (Gonseth et al., 2015). Gene mutations in methylene–tetrahydrofolate reductase and methionine synthase reductase lead to a folate metabolic disturbance. Reduced folic acid uptake causes the down-regulated expression of transcription factor AP-2 alpha, which may increase the level of DNA methylation in the fetus. All of these are likely genetic risk factors that affect the incidence of neural tube malformation.

The establishment and improvement of disease animal models are important for revealing the pathogenesis of human spina bifida, and play key roles in testing novel interventions (Smith et al., 1978; Duru et al., 2001; Danzer et al., 2005). Recent years, researchers have explored the effects of some risk factors by using these animal models (Zhao et al., 2008; Qin et al., 2016), and take further measures such as gene and cell therapy strategies to intervene to reduce the occurrence of perinatal birth defects (Melo-Filho et al., 2009; Turner et al., 2013; Wei et al., 2020a). It is indicated that genetic therapy may become a novel strategy for treating birth defects. Therefore, it is necessary to analyze the gene expression profiles of fetuses with neural tube defects (NTDs), which allows us to further understand the genetic determinants and epigenetic factors involved in this pathological mechanism. Nagy and colleagues (Nagy et al., 2006) contributed a dataset of whole genome mRNA expression profiles from amniocytes in amniotic fluid samples of nine people (GSE4182: five spina bifida and four healthy control samples). They revealed three novel candidate genes: Src like adaptor (*SLAP*), leukocyte specific transcript 1 (*LST1*), and mal, T cell differentiation protein like (*BENE*), which can play an important role in the pathogenesis of NTDs.

However, more than a decade has passed since their results were reported, and many new and effective bioinformatics methods have appeared. Many questions are worthy of further exploration such as a study on gene co-expression modules in human amniocytes from patients with spina bifida. In this study, we used genes in a GSE4182 dataset that were in the top 25% of variance from spina bifida and healthy control amniocytes to construct a co-expression network by WGCNA. We attempted to identify promising candidate biomarkers or potential therapeutic targets of spina bifida from modules in which highly correlated genes clustered. Furthermore, a study of specific molecular and biological functions of these hub genes may be better for understanding the underlying mechanisms of the disease. Based on our previous findings of diagnostic miRNAs in the serum of pregnant women with fetuses that have NTDs, we reveal the candidate genes in a hub module and a miRNA–mRNA interaction network for spina bifida.

## Materials and Methods

### Microarray Dataset Collection

The Gene Expression Omnibus (GEO) database^1^ was used to obtain gene expression profiles for spina bifida. Exclusion criteria: (1) Profiles based on cell lines and animal models were excluded. Inclusion criteria: (1) Only homo sapiens species samples with spina bifida and healthy controls were included in this study. The GSE4182 dataset was detected on a platform of Affymetrix GeneChip Human Genome U133 Plus 2.0 [HG-U133_Plus_2]. GSE4182 included nine amniotic fluid samples from pregnant women with spina bifida (*N* = 4) or healthy (*N* = 5) fetuses. Amniocytes in the amniotic fluid were collected by amniocentesis, from which fetal mRNA was isolated and analyzed. In this study, the expression profile was acquired directly from a public database.

### Data Preprocessing

The RAW data of the expression dataset, GSE4182, in .CEL format were obtained from a GEO database. The “affy” package in R was used to conduct the normalization and background correction of data. Probe level data were then converted into gene expression values. For multiple probes corresponding to a gene, the average expression value was taken as the gene expression value in this study. The distribution patterns of disease and control samples (before and after clustering analysis and outliers removement) were observed by principal component analysis (PCA).

### Identification of Differentially Expressed Genes in Spina Bifida

The “limma” package in R was used to obtained Differentially Expressed Genes (DEGs) between spina bifida and healthy control samples in the expression data. We then carried out a significance analysis of microarrays and set the selection criteria as a false discovery rate (FDR) value <0.05 and log2| fold change| >1 (fold change >2) for further network construction.

### Construction of Co-expression Network

The “WGCNA” package in R was used to construct the co-expression network based on the expression data profile of these top 25% variant genes. The microarray quality was checked by the “impute” package in R, which could detect whether the genes had missing values and ensure they were good samples. We performed sample clustering to plot the sample tree, and to detect and delete outliers. We then performed Pearson’s correlation matrices for pair-wise genes and found a soft thresholding power β value by using the pickSoftThreshold function of WGCNA.

### Identification of Hub Genes

First, we defined the module with the largest absolute value of Pearson’s correlation of module membership (MM) and a *p*-value < 0.05 as the hub module. Furthermore, we defined hub genes in co-expression networks as genes that satisfy two conditions: the absolute value of the Pearson’s correlation of MM >0.8 and the absolute value of the Pearson’s correlation of gene trait (GS) relationship >0.2, which represented high module connectivity and high clinical significance, respectively. We subsequently obtained real hub genes by taking the intersection of hub genes in a co-expression network and significantly DEGs. On the basis of this, such real hub genes were used to construct an miRNA–mRNA regulatory network.

### Functional Enrichment Annotation

Gene Ontology (GO) and Kyoto Encyclopedia of Genes and Genomes (KEGG) pathway analyses of real hub genes were performed by using the online program: Database for Annotation, Visualization, and Integrated Discovery (DAVID) (Dennis et al., 2003). The GO terms were divided into biological process (BP), cellular component (CC), and molecular function (MF). Statistical significance was considered as a *p*-value < 0.05.

### Prediction of miRNA Target Genes and Construction of miRNA–mRNA Regulatory Network

TargetScan^2^ is a miRNA target prediction database that is used for mammals by searching for the presence of conserved 8, 7, and 6 mer sites that match the seed region of each miRNA (Agarwal et al., 2015). As a database, miRTarBase^3^ contains more than 360,000 miRNA–target interactions that have been validated experimentally by reporter assay, western blot, microarray, and high-throughput sequencing (Chou et al., 2018). These two online tools were used to predict target mRNAs in our previous study of diagnostic miRNAs (miR-142-3p, miR-144, miR-720, miR-575, and miR-765) in the serum of pregnant women with fetuses that had spina bifida (Gu et al., 2012). We took the intersection of predicted target mRNAs and real hub genes, and used the results to construct an miRNA–mRNA regulatory network. Furthermore, the network was visualized by Cytoscape 3.7.1 (Shannon et al., 2003).

### Samples Collection

The samples used in this study are from the sample bank of our department, which were collected as previously described (Zhang H. et al., 2019). The study was approved by the Ethics Committee of Shengjing Hospital, China Medical University. The ethics number is 2015PS264K. All consent was obtained to use the samples for testing. Six cases of spinal cord tissue were obtained from fetuses induced labor by spina bifida, and eight cases of spinal cord tissue were obtained from fetuses induced labor by non CNS congenital malformations which were approximately similar age used as normal controls, as shown in the Supplementary Table 1. Tissue samples from spina bifida and healthy control fetuses were collected at pregnancy termination by experienced pathologists and stored at −80°C until analysis.

### Quantitative Real Time Polymerase Chain Reaction

Subsequently, quantitative real time polymerase chain reaction (qRT-PCR) was used to verify the expression of the key genes in spinal cord tissue of clinical samples. Total RNA was isolated from each sample using Invitrogen (15596026) Trizol reagent by our research team member according to the manufacturer’s instructions. Reverse transcription from total RNA to cDNA and qRT-PCR were performed using the Takara PrimeScript RT Master Mix (RR036A) and SYBR Green Premix (RR420A), respectively. The results were analyzed using the 2-ΔΔCt method and represented as fold changes, normalized to GAPDH. The PCR primers used in this study were shown in Supplementary Table 2. Statistically significant was considered as the *p*-value < 0.05.

## Results

### Data Preprocessing and DEG Identification

**FIGURE 1:**
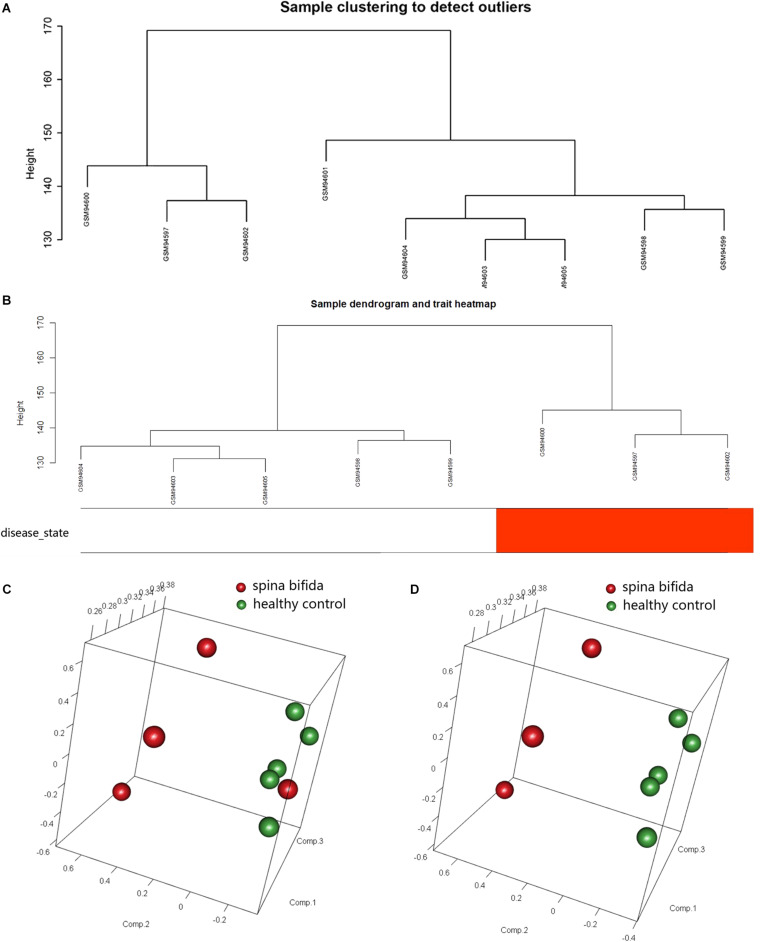
Data Preprocessing. **(A)** Samples clustering of total nine samples in the GSE4182 to detect outliers. **(B)** Re-clustering of the remaining eight samples: sample dendrogram and trait heatmap. The clustering was based on the expression data of DEGs between healthy controls and spina bifida samples. In the disease state, white color means healthy controls and red color means spina bifida. **(C)** PCA for spina bifida and healthy control samples before outlier identification and removal. **(D)** PCA for spina bifida and healthy control samples after outlier identification and removal.

**FIGURE 2:**
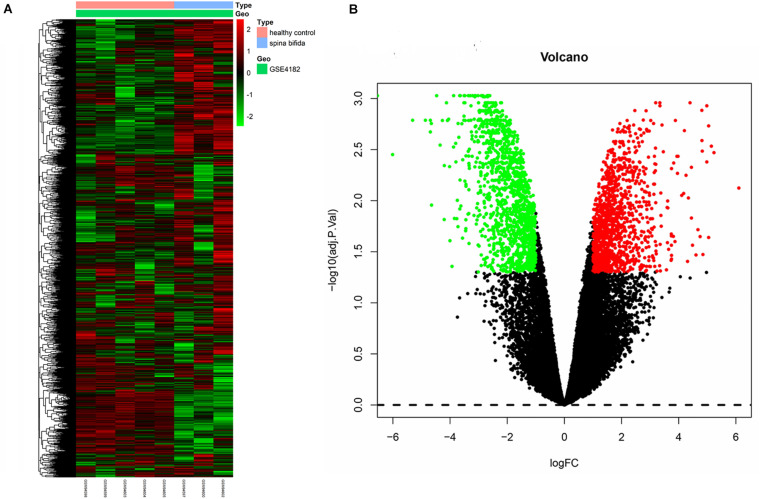
Heatmap and volcano plot of DEGs. **(A)** The heatmap of DEGs. **(B)** The volcano plot of DEGs. Red represents upregulated genes and green indicates down-regulated genes. Black means nondifferentially expressed genes.

The raw data of a GSE4182 dataset that included four spina bifida and five healthy control samples was downloaded. Such data were then preprocessed by conducting format transformation, the filling in of missing data, background correction, and data standardization. The expression matrixes of a total 21,626 genes of these nine samples were obtained. After performing sample clustering to plot the sample tree, it was found that disease sample GSM94601 was clustered with other healthy samples. Then sample GSM94601 was removed as an outlier from a subsequent analysis (Figure 1A). We then matched the disease state of samples with their expression matrixes. The remaining eight samples were re-clustered and a sample dendrogram and trait heatmap were plotted (Figure 1B). At the same time, we performed PCA which showed that samples of spina bifida (except sample GSM94601) were distributed on the left side, while healthy control samples were distributed on the right side. The PCA results were identical with those by clustering analysis. The PCA results before and after outlier removement are shown in Figures 1C,D. The hierarchical cluster analysis heatmap showed significantly different distributions of gene expression patterns between spina bifida fetuses and healthy control samples (Figure 2A). Under the threshold of FDR < 0.05 and fold change >2, a total of 2,634 DEGs (1,277 up-regulated and 1,357 down-regulated) were chosen for further analysis, as shown in Figure 2B.

### Co-expression Network Construction

Based on variance analysis, the top 25% of genes (5,407 genes) was obtained from GSE4182 with eight samples. These 5,407 genes were further analyzed and screened using WGCNA. We explored the value of the weight parameter β of an adjacency matrix by setting up a set of soft-thresholding powers (from 0 to 20). In this study, the soft-thresholding power was chosen as β = 8 where the curve first reached R^2 = 0.88, to construct a weighted network based on a scale-free network distribution (Supplementary Figures 1A–D).

**FIGURE 3:**
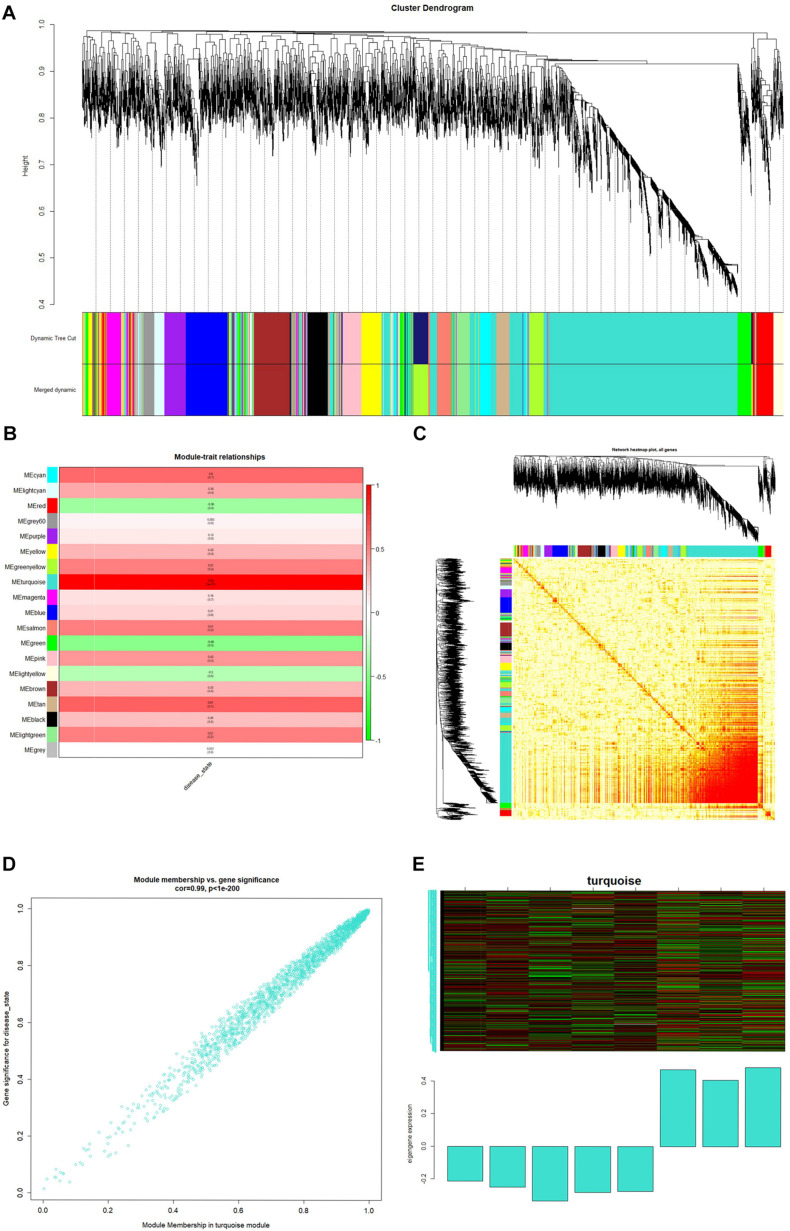
**(A)** Dendrogram of the top 25% of genes clustered based on a dissimilarity measure (1-TOM). **(B)** Heatmap of the correlation between module eigengenes and spina bifida. **(C)** Genetic network heatmap based on topological overlap. **(D)** Scatter diagrams for module membership vs. gene significance of disease state in turquoise modules. **(E)** Heatmap of gene expression in the module and feature vector histogram.

The minimum number of genes was set as 30 for each module, and a clustering of module eigengenes obtained. We then calculated the dissimilarity coefficients between the genes and plotted the system cluster tree. Modules with a similarity above 70% were merged (Supplementary Figure 2A), and a dynamic tree dendrogram was redrawn (Figure 3A). Finally, a total of 19 modules was obtained using a dynamic tree-cutting method. The number of genes in each module is listed in Table 1.

**TABLE 1:** The number of genes in the 19 modules.

Module colors	Number
Black	200
Blue	348
Brown	282
Cyan	172
Green	225
Greenyellow	313
Gray	4
Grey60	133
Lightcyan	136
Lightgreen	123
Lightyellow	118
Magenta	183
Pink	194
Purple	177
Red	207
Salmon	173
Tan	175
Turquoise	1996
Yellow	248

### Identification of Clinically Significant Module

After relating modules to traits, high correlations were observed in traits of the disease state (healthy control or spina bifida) in the turquoise module (Supplementary Figures 2B,C). A clinically significant module was defined as a module with a maximum correlation coefficient (meanwhile *p*-value < 0.05). In this study, a turquoise module (correlation coefficient = 0.99, *p* < 1^–200^) was considered a clinically significant module and eligible for further analysis, as shown in Figure 3B. A genetic network heat map was obtained by calculating topological overlap between the top 25% of genes (Figure 3C). Each row and column of the heat map corresponds to a gene, with the darker the color, the higher the topological overlap, and the higher the density of genes. In Figure 3D, scatter diagrams for MM versus gene significance of disease state are shown in the turquoise modules. We then drew a heat map of gene expression in the turquoise module and its feature vector histogram (Figure 3E).

### Identification of Hub Genes

**FIGURE 4:**
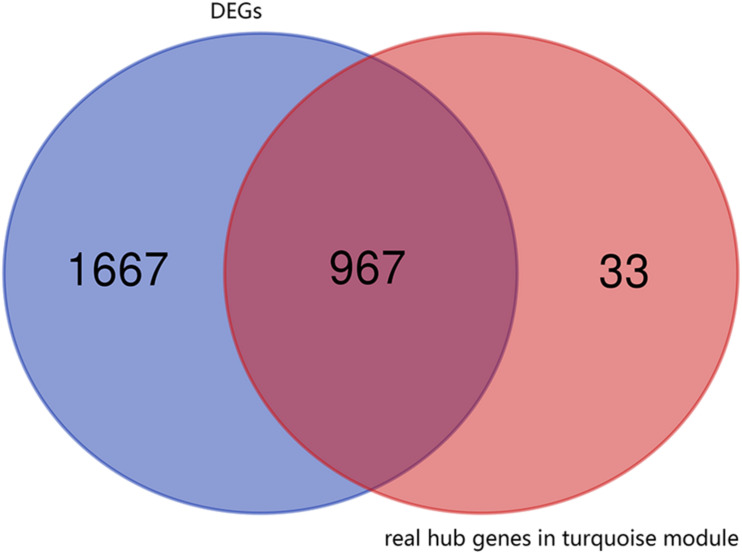
Venn plot of real hub genes in turquoise module and DEGs. The intersection in deep red represents the 967 genes that are common between the real hub genes in the turquoise module and DEGs.

First, the 1000 real hub genes were obtained by screening genes which | GS| >0.2 and | MM| >0.8 in the turquoise module (with total 1996 genes). Then, we overlapped these real hub genes with DEGs with fold change >2 (with total 2634 genes). Finally, the 967 candidate genes (as shown in Figure 4) were obtained for subsequent functional enrichment analysis and miRNA-mRNA network construction.

### Functional Enrichment Annotation

**FIGURE 5:**
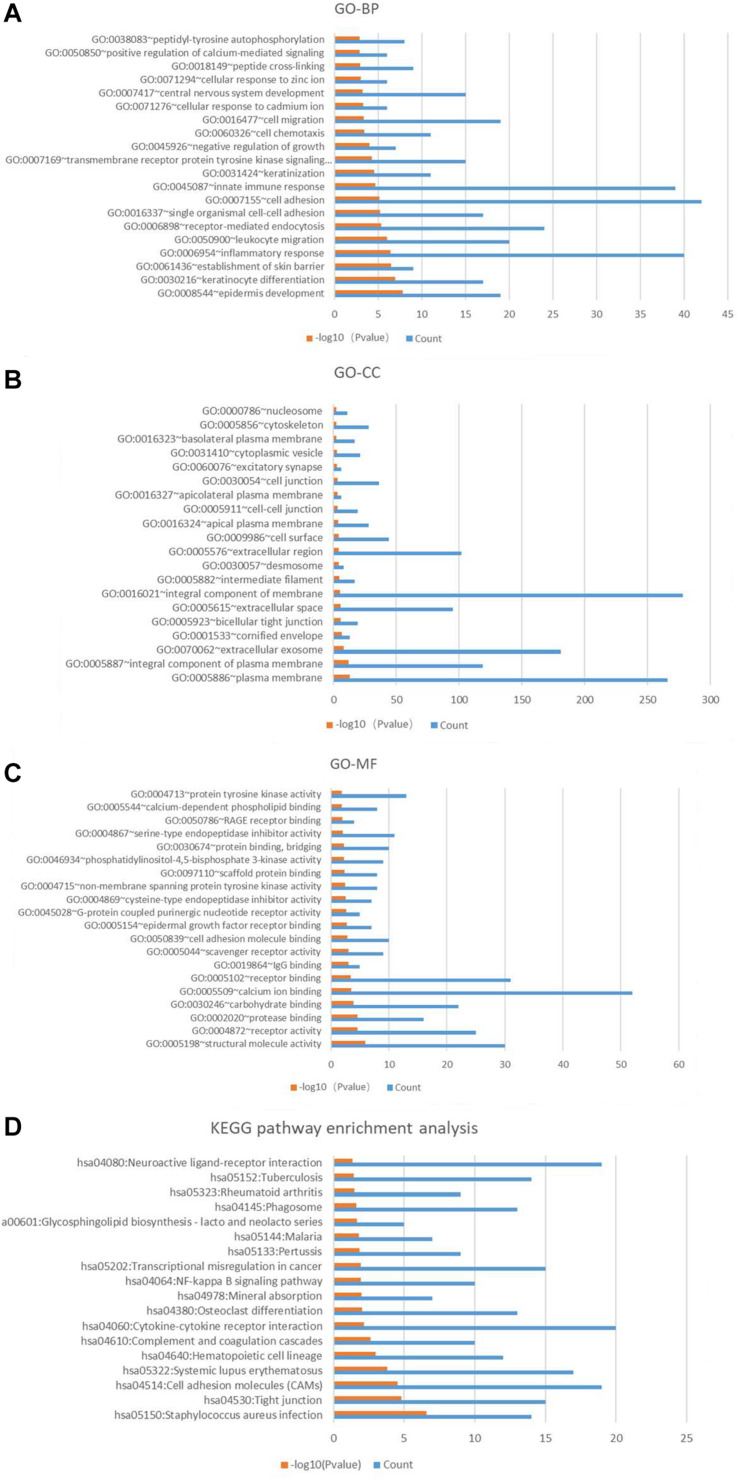
Gene Ontology annotation and pathway enrichment analysis of significantly different genes in turquoise module. **(A)** Top 20 biological process (BP) terms of GO analysis. **(B)** Top 20 cellular component (CC) terms of GO analysis. **(C)** Top 20 molecular function (MF) terms of GO analysis. **(D)** KEGG pathway enrichment analysis. Red color means –log10 (*p*-value), blue color means the gene count.

The results of GO and KEGG analysis of these candidate genes were shown in the Figure 5. It is worth noting that biological processes related to central nervous system development (GO:0007417), cell migration (GO:0016477), keratinization (GO:0031424), innate immune response (GO0045087), inflammatory response (GO:0006954), establishment of skin barrier (GO:0061436), keratinocyte differentiation (GO:0030216), and epidermis development (GO:0008544) were enriched in the top 20 of the results. It may be associated with the skin defect, spinal dysraphism (with or without myelomeningocele) of the spina bifida fetus. The chip samples were collected from amniotic fluid cells, and amniotic fluid cells are derived from fetal exfoliated skin. In a fetus with spina bifida, the edge of the skin of spina bifida may be exfoliated into amniotic fluid and detected. Therefore, the expression of genes in amniotic fluid cells is thought to reflect fetal conditions. The KEGG pathways analysis of these candidate genes indicated that pathways for neuroactive ligand-receptor interaction (hsa04080), NF-kappa B signaling pathway (has04064), osteoclast differentiation (hsa04380), cytokine-cytokine receptor interaction (has04060), and cell adhesion molecules (CAMs) (hsa04514) may play vital roles in the pathological processes of spina bifida.

### Construction of miRNA–mRNA Regulatory Network

**FIGURE 6:**
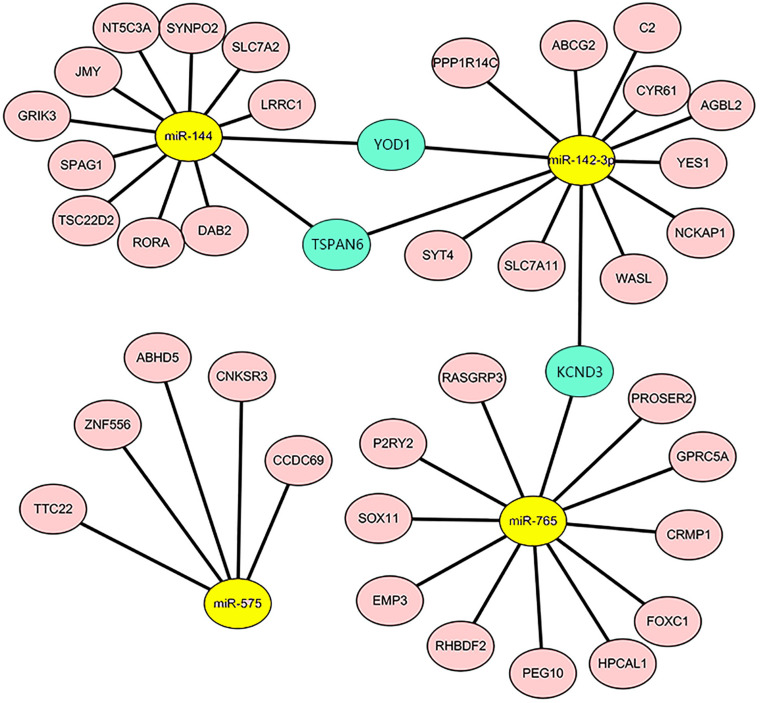
The miRNA-mRNA regulatory network in the spina bifida. The network consisted of four miRNAs (yellow) and 39 mRNAs (light pink). YOD1, TSPAN6, and KCND3 were considered as the three key genes connecting two miRNA gene networks (turquoise).

Our previous study (Gu et al., 2012) explored circulating miRNAs in pregnant women’s sera as potential biomarkers for spina bifida fetuses. We revealed that the expression of five types of miRNAs (miR-142-3p, miR-144, miR-720, miR-575, and miR-765) in the sera of spina bifida fetuses and pregnant women was up-regulated more than two times. Such miRNAs decreased in maternal serum 24 h after delivery, suggesting that maternal serum levels of these five types of dysregulated miRNAs are associated with pregnancy, and which could also reflect fetal conditions to some extent. MicroRNA target genes were predicted by TargetScan and miRTarBase, of which two or more miRNAs might target the same mRNA. The mRNAs appeared simultaneously in the 967 candidate genes and simultaneously predicted ranges were considered as credible mRNAs and shown in the miRNA–mRNA regulatory network (Figure 6). According to the above principles, miRNA-720 did not have credible predicted target genes and was absent in this network. The details of DEGs in credible miRNA–mRNA regulatory networks in spina bifida are shown in Table 2. Three key genes, YOD1, TSPAN6, and KCND3 were, respectively, linked to two miRNA-associated gene networks, of which their potential biological function and underlying mechanism are worthy of further discussion and research.

**TABLE 2:** DEGs in the confirmed miRNA-mRNA regulatory networks.

Up-/Downregulated	Entrez ID	Gene symbol	Gene title	logFC	adj. *P*-Value
Downregulated	220709_at	ZNF556	Zinc finger protein 556	–4.47162	0.000939
	206277_at	P2RY2	P2Y purinoceptor 2	–3.74581	0.009906
	210117_at	SPAG1	Sperm-associated antigen 1	–3.09564	0.002366
	201289_at	CYR61	Cysteine-rich, angiogenic inducer, 61	–2.97183	0.001635
	225720_at	SYNPO2	Synaptopodin-2	–2.92121	0.005667
	215150_at	YOD1	YOD1 deubiquitinase	–2.91952	0.005392
	226682_at	RORA	Nuclear receptor ROR-alpha	–2.85663	0.000939
	212886_at	CCDC69	Coiled-coil domain-containing protein 69	–2.85316	0.001761
	213805_at	ABHD5	1-acylglycerol-3-phosphate O-acyltransferase ABHD5	–2.83363	0.001323
	225516_at	SLC7A2	Cationic amino acid transporter 2	–2.80655	0.01017
	209108_at	TSPAN6	Tetraspanin-6	–2.70781	0.001644
	218816_at	LRRC1	Leucine-rich repeat-containing protein 1	–2.68555	0.001761
	220390_at	AGBL2	Cytosolic carboxypeptidase 2	–2.66653	0.00969
	215106_at	TTC22	Tetratricopeptide repeat domain containing	–2.59286	0.001308
	229801_at	PROSER2	Proline and serine rich 2	–2.49026	0.001181
	226907_at	PPP1R14C	Protein phosphatase 1 regulatory subunit 14C	–2.46988	0.021354
	235563_at	GPRC5A	Retinoic acid-induced protein 3	–2.42726	0.001761
	210953_at	TSC22D2	TSC22 domain family member 2	–2.35737	0.001754
	209921_at	SLC7A11	Cystine/glutamate transporter	–2.31213	0.010217
	210917_at	YES1	Tyrosine-protein kinase Yes	–2.28355	0.006637
	212092_at	PEG10	Retrotransposon-derived protein PEG10	–2.18781	0.014291
	223298_s_at	NT5C3A	Cytosolic 5′-nucleotidase 3A	–2.1847	0.01104
	224813_at	WASL	Neural Wiskott-Aldrich syndrome protein	–2.17078	0.00328
	226352_at	JMY	Junction-mediating and -regulatory protein	–2.16481	0.001791
	213260_at	FOXC1	Forkhead box protein C1	–2.11452	0.002497
	227481_at	CNKSR3	Connector enhancer of kinase suppressor of ras 3	–2.04366	0.005785
Upregulated	209734_at	NCKAP1L	Nck-associated protein 1-like	1.540901	0.003268
	209735_at	ABCG2	ATP-binding cassette sub-family G member 2	1.891656	0.022481
	211301_at	KCND3	Potassium voltage-gated channel subfamily D member 3	2.10085	0.02812
	219202_at	RHBDF2	Inactive rhomboid protein 2	2.159764	0.014825
	203729_at	EMP3	Epithelial membrane protein 3	2.258525	0.001974
	212552_at	HPCAL1	Hippocalcin-like protein 1	2.27348	0.003354
	203052_at	C2	Complement component 2	2.324313	0.012057
	244526_at	RASGRP3	Ras guanyl-releasing protein 3	2.394329	0.006312
	223529_at	SYT4	Synaptotagmin-4	2.695291	0.006394
	204915_s_at	SOX11	Transcription factor SOX-11	2.737709	0.014825
	207454_at	GRIK3	Glutamate receptor ionotropic, kainate 3	2.885433	0.012153
	232898_at	DAB2	Disabled homolog 2	3.180265	0.025146
	202517_at	CRMP1	Collapsin response mediator protein 1	5.04684	0.001854

### Validation in the Clinical Samples

**FIGURE 7:**
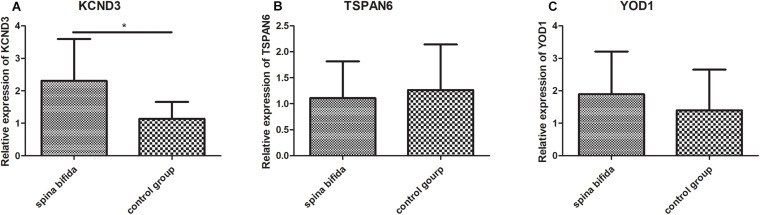
Validation in the clinical samples by qRT-PCR. KCND3 **(A)** upregulated in spina bifida samples. TSPAN6 **(B)** and YOD1 **(C)** did not show significantly statistical difference between two groups. *p < 0.05 compared to the control group.

To further validate the results of the microarray analysis, we examined the expression of the three key genes which have dysregulated expression in spina bifida by qRT-PCR using spinal cord samples from spina bifida and control fetus with induced labor with non CNS congenital malformations. Subsequently, we found that KCND3 and YOD1 were upregulated and TSPAN6 was downregulated in spina bifida samples. The unpaired *t*-test was used for statistical analysis of the data shown in Figure 7. However, only the difference of KCND3 between the two groups had statistical significance (*p* < 0.05), which was consistent with our previous analysis. TSPAN6 had the same decreased expression trend with our bioinformatics analysis results, but did not show a statistically significantly difference between the two groups. However, the changing trend of YOD1 was in the opposite direction.

## Discussion

Spina bifida is one of the most common serious birth defects found worldwide for which prenatal treatment options remain limited. Researchers have thus recognized the urgency for improvements in early preclinical diagnosis and treatment levels. In recent years, our research group has been devoted to the study of the genetic and epigenetic etiology and pathogenesis of spina bifida (Fan et al., 2011; Shan et al., 2012; Wei et al., 2013; Zhang H. et al., 2019; Zhang H.N. et al., 2019; An et al., 2020; Liu et al., 2020), as well as the diagnosis and prenatal diagnosis of diseases (Gu et al., 2012; An et al., 2015), gene therapy (Ma et al., 2020) and stem cell therapy (Li et al., 2012; Wei et al., 2020a,b) and other etiological treatments, which are conducive to improving the level of disease prevention and treatment. However, we still believe more needs to be done. In this study, WGCNA was used to explore pathological processes and marker genes in amniotic fluid cells of the spina bifida fetus. In this study, we identified a miRNA–mRNA regulatory network that consisted of four miRNAs and 39 mRNAs. *TSPAN6*, *YOD1*, and *KCND3* are considered key genes in this network.

As a member of the tetraspanin family, tetraspanin-6 (TSPAN6) was predicted to be co-regulated by miR-142-3p and miR-144 in spina bifida. Guix and colleagues (Guix et al., 2017) reported that TSPAN6 was increased in Alzheimer’s disease brains and promoted Aβ-peptide accumulation by affecting the autophagosome–lysosomal pathway and slowing down the degradation of Amyloid precursor protein (APP)–C-terminal fragments. At the same time, TSPAN6 increased exosome-mediated secretion of APP–C-terminal fragments. TSPAN6 was found to be important for cognition and to affect properties of the postsynaptic terminal (Salas et al., 2017). Deletions at TSPAN6 cause epilepsy female-restricted with intellectual disability (Vincent et al., 2012). In addition, TSPAN6 affected mitochondrial antiviral signaling (MAVS) formation in a ubiquitination-dependent manner, which further negatively regulated the retinoic acid–inducible gene I-like receptor (RLR) pathway and host antiviral immune response (Wang et al., 2012). In general, all-trans retinoic acid was employed as a spina bifida aperta–inducing agent by our research group (Xue et al., 2018; Zhang H.N. et al., 2019) and other international peers (Oria et al., 2018). TSPAN6 may be tractable as a therapeutic target for spina bifida based on the results obtained from a literature search and data analysis.

As a gene encoding deubiquitinating enzyme, YOD1 was predicted to be co-regulated by miR-142-3p and miR-144 in spina bifida. Previous studies suggested that YOD1 correlated with another congenital malformed disease: nonsyndromic cleft lip, with or without cleft palate (NSCL/P). RNA interference and overexpression experiments indicated that YOD1 could enhance cell migration during lip and palate formation through the transforming growth factor (TGF)-β3 signaling pathway (Zhou et al., 2018). A mutation of YOD1 may lead to impeded cell migration resulting in NSCL/P (Ju et al., 2018). We therefore have reason to speculate that YOD1 plays a similar role in spina bifida. A few studies have highlighted the significant role of YOD1 in neurodegenerative disease. Tanji et al. demonstrated that the neurogenic proteins that cause Huntington and Parkinson’s diseases induced upregulation of the YOD1 level (Tanji et al., 2018). Ubiquitin-directed AAA-ATPase p97 cooperating with YOD1 played an essential role in the clearance of ruptured lysosomes by autophagy, in which a mutation caused inclusion body myopathy and neurodegeneration (Papadopoulos et al., 2017). Overall, such data suggest that the deubiquitinase YOD1 contributes to the pathogenesis of neurodegenerative disease by affecting the ubiquitin–proteasome system and autophagy–lysosome pathway. Data has also suggested that YOD1 plays an important role in the development of an antiviral immune response. Liu and colleagues reported YOD1-mediated K63-linked deubiquitination could activate an innate antiviral immune response against viral infection, and the aggregation of MAVS (Liu et al., 2019). In addition, a catalytically inactive mutant of the deubiquitinase YOD1 enhances antigen cross-presentation (Sehrawat et al., 2013). YOD1 antagonizes ubiquitin ligase TRAF6/p62-dependent interleukin-1 signaling to NF-κB (Schimmack et al., 2017). Past studies have suggested that YOD1 and TSPAN6 both affect MAVS formation and an active antiviral immune response by regulating the ubiquitination pathway. More speculatively, spina bifida may be related to a type of viral infection.

As a gene encoding potassium voltage-gated channel subfamily D member 3, *KCND3* was predicted to be co-regulated by miR-142-3p and miR-765 in spina bifida. It was shown that a mutation in *KCND3* was associated with the autosomal dominant inherited neurodegenerative disorder, spinocerebellar ataxia types 19 and 22 (SCA19/22) (Duarri et al., 2012; Lee et al., 2012; Paucar et al., 2018; Hsiao et al., 2019). Moreover, KCND3 pathogenic variants may be responsible for a wider phenotypic spectrum than previously thought, including autosomal recessive early-onset sporadic cerebellar ataxia (Kurihara et al., 2018), childhood epileptic encephalopathy (Wang et al., 2019), Parkinsonism, and cognitive impairment (Huin et al., 2017). However, further experimental studies are needed to validate a dysfunction of the potassium voltage-gated channel exists in spina bifida.

In this study, novel biomarkers of spina bifida have been provisionally identified from the viewpoint of coexpression network analysis. Our findings also provide novel insights into an miRNA–mRNA regulatory network in spina bifida. The genes we identified as key genes were derived from the hub module (with highest correlation with disease, shown in Figure 3B) by WGCNA. Furthermore, the miRNAs we used for constructing the miRNA-mRNA network were obtained from our previous verified dysregulated microarray results in spina bifida. Based on our preliminary experimental validation results, KCND3 was upregulated in the spina bifida group, which supports that KCND3 may play a role in the development of spina bifida. This result corresponded to that of our bioinformatics analysis. According to Figure 6, miR-765 and miR-142-3p may play a role by regulating the expression of KCND3 involved in the development of spina bifida. But how they participate in the disease onset remains unclear. These details need to be confirmed by further in depth experimental studies, and this will be an important research direction to uncover the molecular mechanisms of abnormal expression of these gene and miRNAs in spina bifida. Furthermore, future research and validation studies need to be based on larger sample sizes.

## Conclusions and Future Prospects

The incidence of NTDs is still high in some underdeveloped countries and regions. According to reports, folic acid–preventable spina bifida–related stillbirths and child mortalities are still widespread in Ethiopia (Dixon et al., 2019). Recently, our team and international peers have reported that *in utero* amniotic fluid stem cell therapy techniques may promised new hope to sufferers of NTDs (Abe et al., 2019; Huang et al., 2020). Our research group has made some important improvement in the field of transamniotic bone marrow mesenchymal stem cell transplantation therapy as well (Wei et al., 2020a,b). In this study, we identified a miRNA–mRNA regulatory network including four miRNAs and 39 mRNAs in spina bifida and implicate *TSPAN6*, *YOD1*, and *KCND3*, ubiquitylation pathways, and an antiviral immune response as modulators of neural tube malformations. Following the verification of qRT-PCR and KCND3 was upregulated in spina bifida. KCND3 and its related miR-765 and miR-142-3p appear worthy of further study. However, it remains unclear whether amniocytes reflect the genetic, epigenetic and/or environmental influences related to spina bifida. Such findings and proposed mechanisms obtained from our bioinformatics analyses require validation in spinal cord and adjacent tissues from spina bifida samples by further experimental research using larger sample sizes.

## Data Availability Statement

Raw data is available at GEO database under accession number GSE4182 here: https://www.ncbi.nlm.nih.gov/geo/query/acc.cgi?acc=GSE4182. The data used to support the findings of this study are available from the corresponding author upon request.

## Ethics Statement

The studies involving human participants were reviewed and approved by Ethics Committee Shengjing Hospital of China Medical University (No. 2015PS264K). Written informed consent to participate in this study was provided by the participants’ legal guardian/next of kin.

## Author Contributions

ZL analyzed the data, wrote the manuscript, and created the figures and tables. As the postdoctoral co-supervisors of ZL, JF, and ZY provided critical revisions and conceptual support. All authors approved of the final submission.

## Conflict of Interest

The authors declare that the research was conducted in the absence of any commercial or financial relationships that could be construed as a potential conflict of interest.

## Footnotes

1 http://www.ncbi.nlm.nih.gov/geo

2 http://www.targetscan.org/vert_72/

3 http://mirtarbase.mbc.nctu.edu.tw/

## Supplementary Material

Supplementary Figure 1 Determination of soft-thresholding power in the WGCNA. **(A)** Analysis of the scale-free index for a set of soft-thresholding powers (β). **(B)** Analysis of the mean connectivity for a set of soft-thresholding powers. **(C)** Histogram of connectivity distribution when β = 8. **(D)** Checking the scale free topology when β = 8.

Supplementary Figure 2 **(A)** Screening of modules needed to be merged. **(B)** Cluster dendrogram of relation between modules and clinical traits. **(C)** Clustering heatmap of relation between modules and clinical traits.

Supplementary Table 1 Summary of clinical features of samples in this study.

Supplementary Table 2 Sequences of primers used for qRT-PCR.
