# Supplementary figures and images for: Key Modules and Hub Genes Identified by Coexpression Network Analysis for Revealing Novel Biomarkers for Spina Bifida
Source: Front Genet. 2020 Dec 2;11:583316. doi: 10.3389/fgene.2020.583316 (PMC7738565; doi:10.3389/fgene.2020.583316)

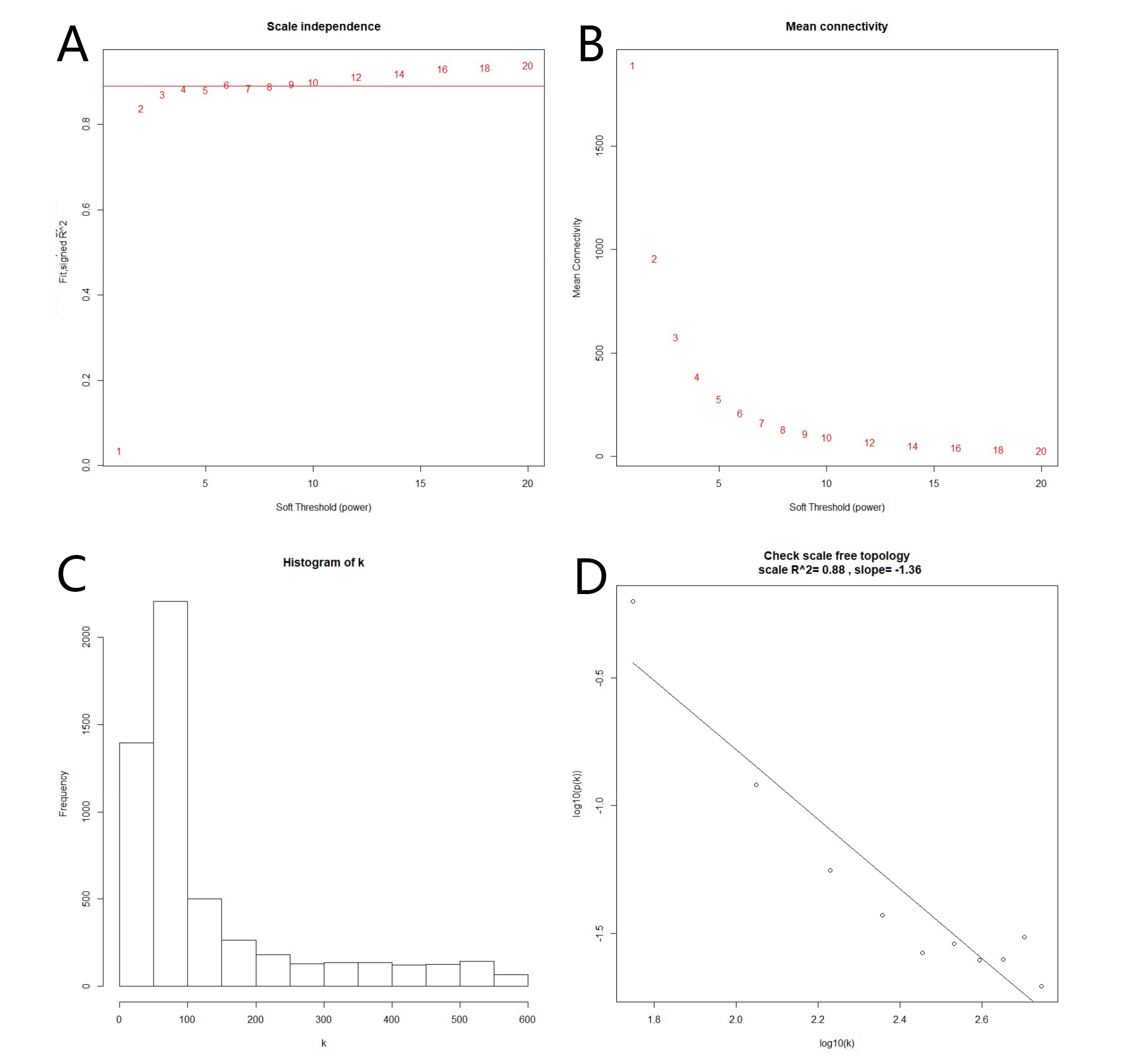

Supplement: Supplementary Figure 1 — Determination of soft-thresholding power in the WGCNA. (A) Analysis of the scale-free index for a set of soft-thresholding powers (β). (B) Analysis of the mean connectivity for a set of soft-thresholding powers. (C) Histogram of connectivity distribution when β = 8. (D) Checking the scale free topology when β = 8. [file Image_1.TIF]

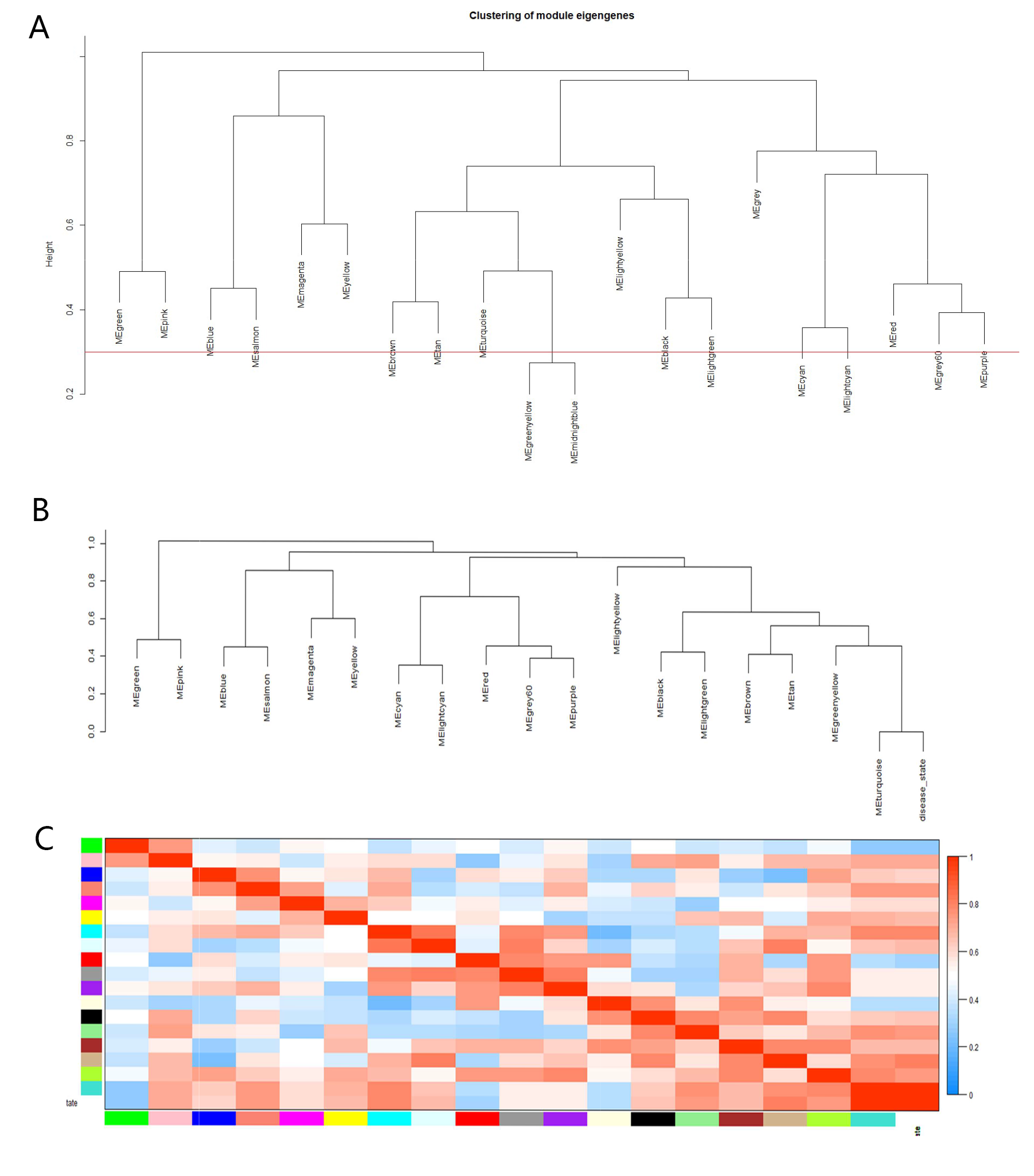

Supplement: Supplementary Figure 2 — (A) Screening of modules needed to be merged. (B) Cluster dendrogram of relation between modules and clinical traits. (C) Clustering heatmap of relation between modules and clinical traits. [file Image_2.TIF]
