# Supplementary material for: Key Modules and Hub Genes Identified by Coexpression Network Analysis for Revealing Novel Biomarkers for Spina Bifida
Source: Front Genet. 2020 Dec 2;11:583316. doi: 10.3389/fgene.2020.583316 (PMC7738565; doi:10.3389/fgene.2020.583316)
Supplement: Supplementary Table 1 — Summary of clinical features of samples in this study. [file Table_1.DOCX]

| **Supplementary Table 1. Summary of clinical features of samples in this study.** | | | | |
| --- | --- | --- | --- | --- |
| Group | Sample label | Types of deformity | Sex | Gestational weeks |
| Spina bifida group | ES1 | Meningomyelocele | F | 26 weeks |
|  | S1(ES3) | Spina bifida | F | 29 weeks+3 days |
|  | ES4 | Spina bifida | F | 27 weeks+6 days |
|  | S5(ES7) | Spina bifida | M | 33 weeks |
|  | ES8 | Spina bifida,hydrocephaly | F | 30 weeks+6 days |
|  | ES9 | Spina bifida | F | 24 weeks |
| Control group | N1(807416) | Inevitable abortion | F | 28 weeks+6 days |
|  | N2(707666) | Mild pericardial effusion | F | 30 weeks+3 days |
|  | S189 | Inevitable abortion | F | 26 weeks+6 days |
|  | S90 | Unplanned pregnancy | M | 34 weeks |
|  | S108 | Inevitable abortion | F | 25 weeks+6 days |
|  | S109 | Inevitable abortion | F | 25 weeks+6 days |
|  | S169 | Unplanned pregnancy | M | 26 weeks+4 days |
|  | S168 | Threatened premature labor | M | 29 weeks+4 days |
|  |  |  |  |  |

| **Supplementary Table 2. Sequences of primers used for qRT-PCR.** | | | |
| --- | --- | --- | --- |
| Gene | Gene bank accession | Chains | Sequence |
| YOD1 | NM_018566 | forward | 5’-GCCCATCCAATCTGGTGACA-3’ |
|  |  | reverse | 5’-CACGGTTCTGGTAAGCACAG-3’ |
| TSPAN6 | NM_003270 | forward | 5’-TCCACAGAGAGATGCAGACA-3’ |
|  |  | reverse | 5’-TCCAATCAGTTGGAAGCAAGC-3’ |
| KCND3 | NM_172198 | forward | 5’-TCCACCATCAAGAACCACGA-3’ |
|  |  | reverse | 5’-TCTTACTACGACGGGAGCAG-3’ |
| GAPDH | NM_002046 | forward | 5’-GCACCGTCAAGGCTGAGAAC-3’ |
|  |  | reverse | 5’-TGGTGAAGACGCCAGTGGA-3’ |
